# On the Ground or in the Air? A Methodological Experiment on Crop Residue Cover Measurement in Ethiopia

**DOI:** 10.1007/s00267-017-0898-0

**Published:** 2017-06-08

**Authors:** Frédéric Kosmowski, James Stevenson, Jeff Campbell, Alemayehu Ambel, Asmelash Haile Tsegay

**Affiliations:** 1 CGIAR Standing Panel on Impact Assessment, Food and Agriculture Organization of the United Nations, Rome, Italy; 2 Spatial Solutions Inc, Bend, OR USA; 3 The World Bank, Washington, DC USA

**Keywords:** Conservation agriculture adoption, Crop residue coverage, Agricultural remote sensing, Drone, NDTI

## Abstract

Maintaining permanent coverage of the soil using crop residues is an important and commonly recommended practice in conservation agriculture. Measuring this practice is an essential step in improving knowledge about the adoption and impact of conservation agriculture. Different data collection methods can be implemented to capture the field level crop residue coverage for a given plot, each with its own implication on survey budget, implementation speed and respondent and interviewer burden. In this paper, six alternative methods of crop residue coverage measurement are tested among the same sample of rural households in Ethiopia. The relative accuracy of these methods are compared against a benchmark, the line-transect method. The alternative methods compared against the benchmark include: (i) interviewee (respondent) estimation; (ii) enumerator estimation visiting the field; (iii) interviewee with visual-aid without visiting the field; (iv) enumerator with visual-aid visiting the field; (v) field picture collected with a drone and analyzed with image-processing methods and (vi) satellite picture of the field analyzed with remote sensing methods. Results of the methodological experiment show that survey-based methods tend to underestimate field residue cover. When quantitative data on cover are needed, the best estimates are provided by visual-aid protocols. For categorical analysis (i.e., >30% cover or not), visual-aid protocols and remote sensing methods perform equally well. Among survey-based methods, the strongest correlates of measurement errors are total farm size, field size, distance, and slope. Results deliver a ranking of measurement options that can inform survey practitioners and researchers.

## Introduction

In many parts of the world, soil degradation threatens the productive capacity of farmland while demographic pressure limits the potential to farm new lands. In order to achieve increases in agricultural productivity, a better and more sustainable use of land is advocated (sustainable intensification) by proponents of conservation agriculture. Thus, conservation agriculture has received considerable attention among scholars and policy makers (Kassam et al. 2009; Erenstein et al. 2012; Thierfelder and Wall 2012; Tesfaye et al. 2015). The CGIAR—a global partnership on international agriculture research—has invested significantly into conservation agriculture research over the last decades (Renkow and Byerlee 2010) and a growing number of development organizations have been promoting conservation agriculture, with recent efforts focusing on smallholder farming systems in sub-Saharan Africa and Asia (Stevenson et al. 2014).

Conservation agriculture is a set of practices aimed at reducing soil erosion, improving water management and enhancing crop yields. According to the Food and Agriculture Organization (FAO) definition, conservation agriculture is characterized by three crop management practices: (i) minimum mechanical soil disturbance (zero or minimum tillage); (ii) permanent soil cover with crop residues or cover crops, and (iii) diversification of crop species grown in sequences (crop rotation) and/or associations (intercropping). These practices are interlinked but recent evidence from meta-analyses indicate that permanent soil cover is an essential component (Corbeels et al. 2014; Pittelkow et al. 2015).

Conservation agriculture has risen to prominence in the policy discourse on sustainable intensification in spite of a lack of evidence of its adoption by farmers—a problem that is particularly acute in sub-Saharan Africa (Stevenson et al. 2014). As noted by Andersson and D’Souza (2014), considerable variation exists in those adoption estimates that are available and it is not clear how many hectares of land are currently under conservation agriculture. Despite the important implications for policy and resource allocation, very few studies have examined the accuracy of alternative methods to estimate conservation agriculture adoption. One exception is the paper by Kondylis et al. (2015) who found that, in the context of a household survey in Mozambique, questions about adoption of mulching and strip tillage were answered “correctly” (when verified by visits to the plot) by between 85 and 95% of respondents, while the error rate was more pronounced for intercropping (80% correct).

A sufficient condition for adoption of the second pillar of conservation agriculture—crop residue coverage—is where a plot has at least 30% of the soil surface covered by organic material immediately after the planting operation. The 30% threshold is used in international agricultural statistics (OECD 2001; FAO 2016). FAO’s AQUASTAT database goes even further, by distinguishing three categories of adopters: 30–60, 60–90, and >90% ground cover.

Studies of conservation agriculture adoption have been lacking in the literature, and in order to be policy-relevant, adoption estimates should be grounded in nationally representative surveys. Throughout sub-Saharan Africa, several National Statistical Institutes collect data on crop residue use, although not always for the purpose of conservation agriculture measurement. Statistical institutes in Malawi, Niger, Tanzania, and Uganda have measured crop residue use as a binary variable. Other countries, such as Zambia, focus on the main tillage method utilized by farmers, whereas Ethiopia collects data on estimated field residue coverage. Such data are self-reported by the farmer. Assessing whether a condition of 30% minimum crop residue coverage is met could be particularly error-prone using self-reported measures. Furthermore, in the context of econometric analysis that estimates the role that adoption of conservation agriculture has on productivity and other metrics, there is a danger that self-reported measures could be biased—individuals more skilled at farming (something that is typically unmeasured in surveys) could also be more skilled at identifying their adoption status correctly. Therefore, there is a need to identify low-cost, reliable methods for capturing this key element of conservation agriculture.

Low-cost alternative approaches to crop residue cover measurement include the use of visual-aid protocols to approximate the current residue cover, as well as field images or videos analyzed through image-processing methods (Woebbeck et al. 1995). The use of remote sensing technologies has also been tested in the US, and tillage indices have been applied with mixed results (Nagler et al. 2003; Serbin et al. 2009a, b; Daughtry et al. 2010). Although several challenges remain (Zheng et al. 2014), remote sensing technology could represent a huge step forward, by allowing broad-scale mapping of conservation agriculture adoption.

This data capture experiment contributes to a growing literature on agricultural survey methodology (Carletto et al. 2013; Zezza et al. 2016). In order to assess the accuracy of different measurement methods, real survey conditions should be reproduced in an experimental setting. This can be achieved by using a clear benchmark or reference method against which all other methods are compared. In this experiment, a within-plot line-transect benchmark is compared to six lower-cost, alternative methods for estimating crop residue coverage for a plot. The data collection methods under consideration should be able to match the reference distribution of crop residues on the plot as determined by the line transect (LT).^1^

Two hypothesis are explored in this paper. The first expectation is that methods relying on a self-reported estimations fail to capture the indicator of interest. We therefore hypothesize that using a visual-aid protocol depicting different level of residue coverage can help respondents to provide more accurate responses than simply answering an open question. Visual-aid protocols are relatively easy to integrate into existing agricultural surveys and have the potential to overcome language or educational barriers, which may be covariate with estimation error. Second, the field of human vision may limit the accurate measurement of residue coverage over a large area of land. Thus, data collected through ground observations may only be accurate for a small portion of a specific plot that is assessed by human eyes. Consequently, this paper explore the use of aerial data, collected through drones and satellites. We hypothesize that aerial measurement methods perform better than methods based on human observation from the ground.

## Data and Methods

### Survey Experiment

**Fig. 1:**
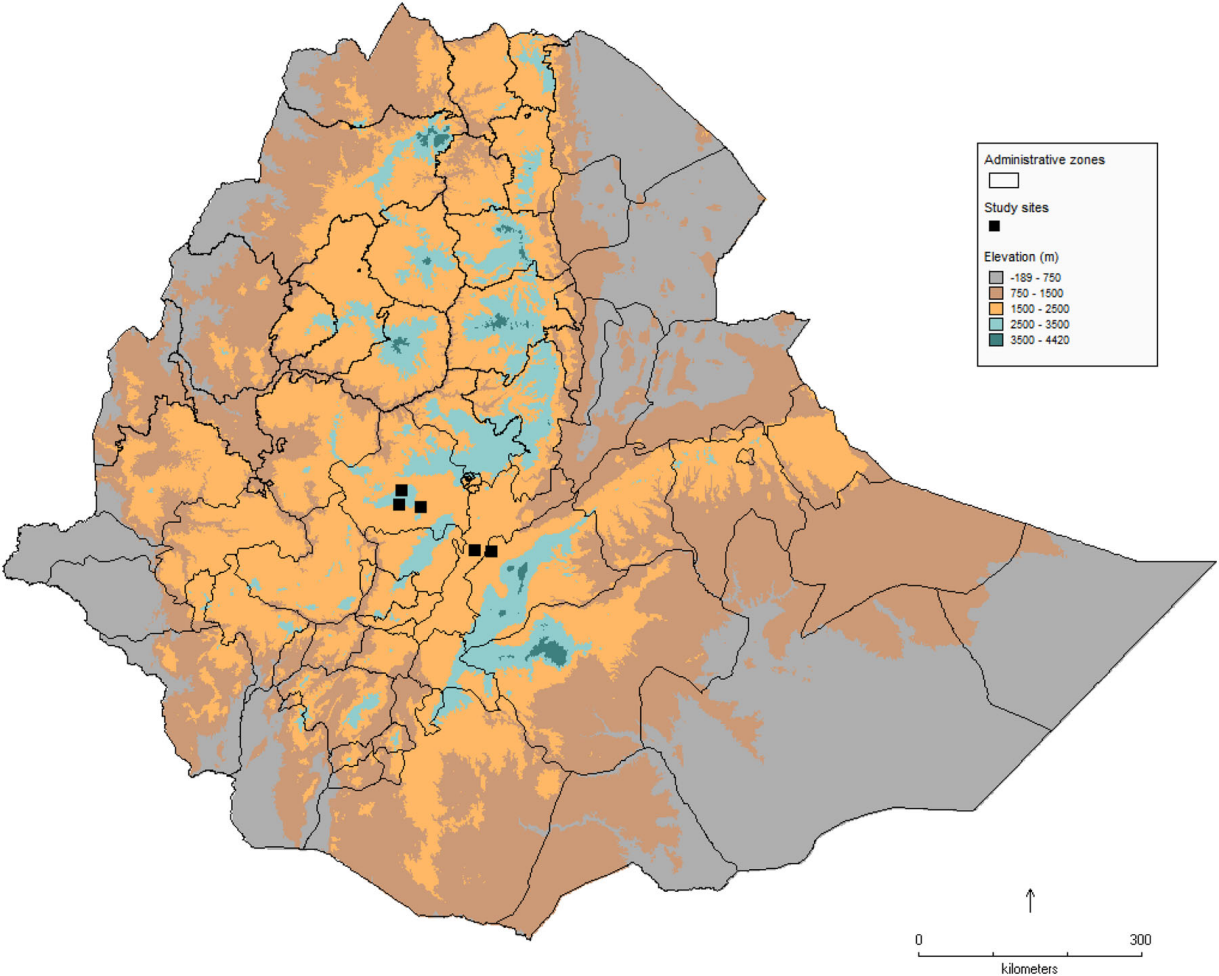
Map of Ethiopia showing the location of study sites in the East and West Shewa zones

The data capture experiment was implemented in five enumeration areas located in the sub-humid areas of East and West Shewa zones in Ethiopia (Fig. 1). The sub-humid agro-ecological areas of Ethiopia are relatively more suitable for the adoption of crop residue cover (Alemu et al. 2006; Tesfaye et al. 2015). In each enumeration area, 12 panel households from the Ethiopian Socio-Economic Survey were interviewed.^2^ In addition, 28 households were randomly selected to participate in the experiment. Data collection took place in December 2015 in East Shewa and February 2016 in West Shewa. Informed written consent was obtained from each household and enumerators were closely supervised, ensuring the collection of high-quality data. This resulted in a total sample of 197 households and 314 plots.

Table 1 provides descriptive statistics of the sample, which is representative of the five enumeration areas. Small-scale agriculture is widely practiced, with an average farm size of 1.2 ha. The sample is well balanced between crop residue types (wheat, maize, barley, teff) and soil types (vertisol, leptosol, luvisol, cambisol). Half of the farmers in the sample indicated that they had received information by extension services on the use of crop residues in the past. However, almost all farmers (95%) in the sample use crop residues as animal feed, while 19% use residues for firewood and 5% use residues for construction purposes^3^. Communal grazing of cropland outside the cropping season is a common practice, limiting the farmers’ ability to completely control the fate of the crop residues on their plots.

**Table 1:** Background statistics of the sampled households and fields

*Household characteristics*	
Household size	5.6
Sex of the head (male)	49.4
Age of the head (years)	46.2
Years of education of the head	3.3
Herd size (in Tropical Livestock Units (TLU))	2.8
Total farm size (ha)	1.2
*Field characteristics*	
Field size (m^2^)	2139
Distance from household (m)	433
Barley residues (%)	22.0
Maize residues (%)	28.0
Teff residues (%)	19.1
Wheat residues (%)	30.9
Cambisol (%)	14.6
Leptosol (%)	25.5
Luvisol (%)	20.7
Vertisol (%)	39.2

Seven methods of crop residue coverage measurement are used in this paper and summarized in Table 2—the LT method and six alternative methods for comparison (M1–6). The survey questionnaire included two modules. Module-1 took place at the interviewee’s home. Tablets equipped with the Open Data Kit application were utilized to collect data on socio-demographic characteristics and farming activities of the household. Fields of maize, wheat, barley and teff were eligible for the experiment and a maximum of two fields were randomly selected for the second module of the survey.^4^ The home-administered Module-1 was then used to collect the respondent estimation (M1) based on recall. This method, applied for example in Jaleta et al. (2015), closely replicates the conditions of typical agricultural household surveys.

**Table 2:** Survey experiment methods

Method	Measurement	Description	(*N*)
LT	Line-transect	Average of four measures taken at the cardinal points of the field	314
M1	Interviewee estimation	Percentage estimation, away from field	314
M2	Enumerator estimation	Percentage estimation, visiting the field	314
M3	Interviewee visual-aid	Identification among six pictures, away from field	314
M4	Enumerator visual-aid	Identification among six pictures, visiting the field	314
M5	Drone image processing	Field picture taken by a drone at a 7.5 m altitude (0.27 cm/pixel resolution) used to segment RGB components	182
M6	Remote sensing	Landsat 8 Thematic Mapper satellite imagery Multispectral (30 m/pixel resolution) used to compute a Normalized Difference Tillage Index (NDTI).	251

The visual-aid protocol (M3, see Fig. A.1) was also presented to respondents at home, who were then invited to identify the photo most closely matching the current state of their eligible plots. To avoid potential bias, the order of M1/M3 and M2/M4 questions was randomized.

Module-2 was completed by the enumerator at the plot, accompanied by the farmer, and methods M2 and M4 were completed in a randomized sequence. Finally, the plots were georeferenced^5^ and a LT was used. Highly applied in agronomy and ecology, the line-transect method is considered a reliable way to determine residue cover (Laflen et al. 1981; Shelton et al. 1995; Kline 2000). A 30 m rope with markings at 1 m intervals was operated by enumerators. First, the LT was laid diagonally on the field’s corners. Then, enumerators were trained to look straight down from directly above each mark and count the number of marks on the rope that intersect over a piece of residue. The operation was repeated at the four corners of the field. These four measures were then averaged to obtain an estimate of residue cover for the entire field. To confirm the reliability of the LT, measures of 20 fields were taken at a 2 month interval. In all cases, the expected pattern of reduction in crop residue cover is observed, with a reduction of 25% on average.

### Drone Image Processing

Low-cost drones (Phantom 2+) were used to capture aerial pictures of the surveyed fields. Since there is a relationship between the altitude required to capture a full image of a field and image resolution, a bias could arise in comparing full field pictures that have different resolutions. Thus, we made the choice to use a unique resolution for all drone aerial pictures and the drones were piloted to take a picture at a 7.5 m altitude from the field center. At this altitude, the image covers approximately 80 m^2^ and provides a resolution of 0.27 cm/pixel.

**Fig. 2:**
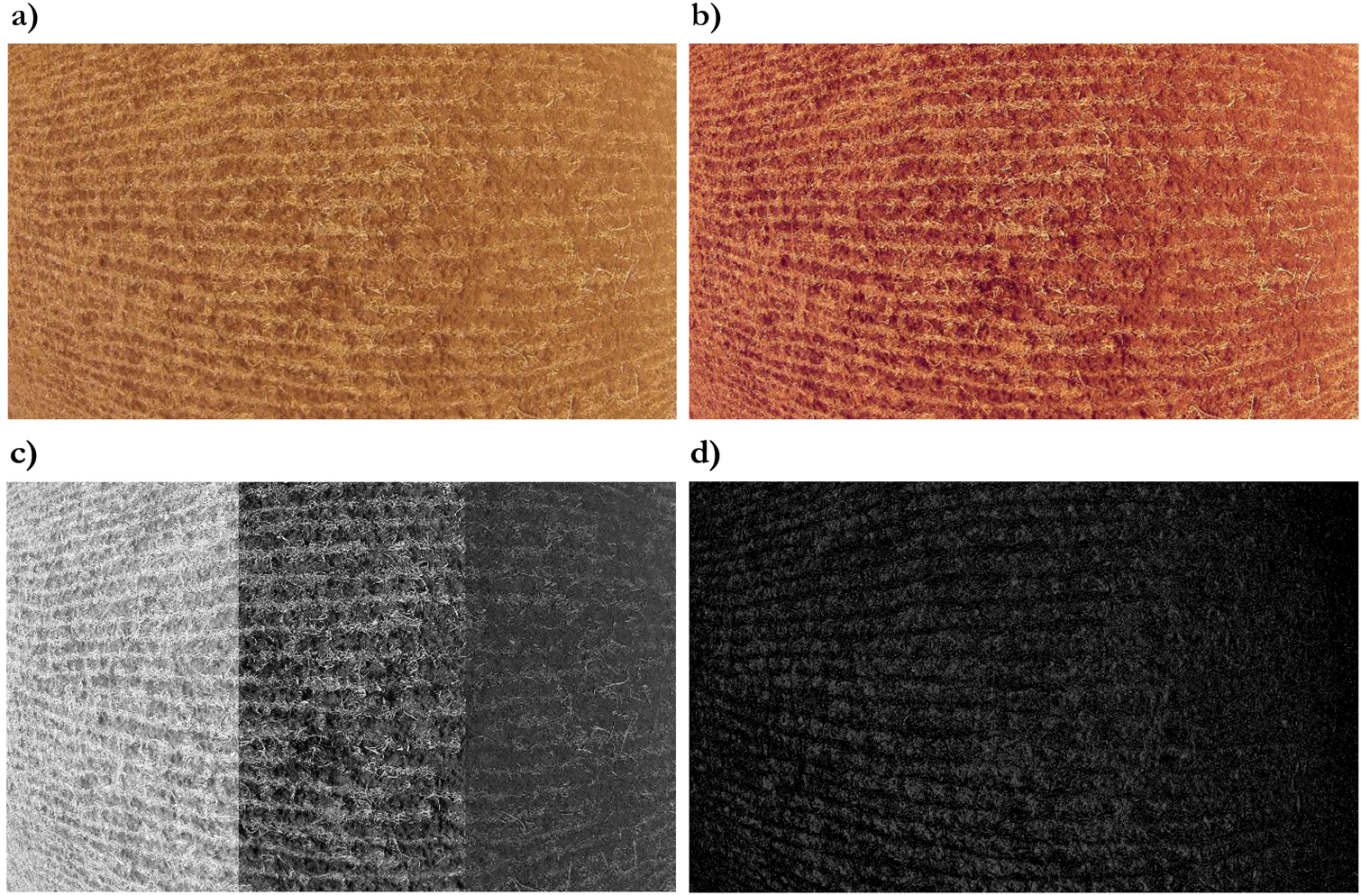
Residue segmentation image processing: **a** original field picture taken by a drone at a 7.5 meter altitude, **b** color balance transformation, **c** extraction of RGB components and **d** segmentation result after application of the 2*G-R-B formula. Soil is represented in black pixels while residues are in white pixels

Image processing techniques are a fast and convenient method for assessing residues on the ground (Woebbeck et al. 1995; Asadi and Jafari 2011). The method of analysis consists of extracting Red-Green-Blue (RGB) components and apply an algorithm to segment the residues from the soil in the images. Image segmentation was performed with the Fiji software (Schindelin et al. 2012) and the steps followed to produce the estimate are presented in Fig. 2. First, a color balance transformation was applied to enhance contrasts between colors. Second, the RGB components of the image were extracted from the full-color image. In order to achieve the segmentation of the residues from the soil, the 2*G-R-B formula was applied as a third step (Asadi and Jafari 2011). The transformation resulted in a binary image with white pixels representing residues and black pixels representing the soil (Fig. 2c). Finally, the percentage of crop residue coverage was determined by dividing the white pixels by the total of pixels from the image.

### Remote Sensing Analysis

Research on mapping conservation practices using remote sensing methods has flourished in recent years. Several indices such as the cellulose absorption index, the lignin-cellulose absorption index, the shortwave IR normalized residue index (SINDRI) or the normalized difference tillage index (NDTI) have been applied with encouraging results (Nagler et al. 2003; Serbin et al. 2009a, b; Daughtry et al. 2010; Zheng et al. 2014). The calculation of these indices rely on various sensor types and bandwidths. Landsat 8 Thematic Mapper (TM) satellite images were chosen because the images are freely available and the satellite has a 16 days revisit interval. Landsat TM images were used to calculate the NDTI, considered to be the best Landsat-based tillage index (Serbin et al. 2009a, b; Zheng et al. 2014).

After survey completion, two archived full scenes of Landsat 8 TM satellite imagery were acquired from the United States Geological Survey’s Earth Explorer imagery search and delivery website. The two full scenes of interest were identified based on their complete coverage of the provided field location coordinates identified above, as well as the plot-based data collection dates associated with each field location (7 December 2015 for East Shewa and 16 February 2016 for West Shewa). Thus, all measurement methods collected during the survey (LT and M1 to M5) refer to the same time as remote sensing measurements. Following the contribution of van Deventer et al. (1997), the NDTI was calculated using the formula:

$${\rm{NDTI}} = {\rm{SWIR1}} - {\rm{SWIR2}}/{\rm{SWIR1}} + {\rm{SWIR2}}$$

The index was then scaled from 0 to 100 for comparison with other measures. The index was not calibrated.

### Data Analysis

Reference results from the line-transect method are used to compare all other methods. Of particular interest in this study is how different measurement methods perform in estimating quantitative vs. categorical measures of crop residue cover. First, the analysis is implemented using quantitative data. Boxplots are used to explore the average estimates provided by each measurement method. To further study the distribution of each measurement method and understand how well they perform along the entire distribution, we employ correlation coefficients and scatterplots. Second, we compare the six methods in terms of how aggregate binary (i.e., yes / no) adoption estimates for the plots meeting the sufficient condition of 30% residue coverage. Following FAO’s AQUASTAT, three categories of adopters: 30–60, 60–90, and >90% ground cover are also distinguished in the analysis for each method. Finally, we use a series of linear probability regressions to estimate measurement errors of adoption of a minimum 30% residue coverage. The dependent variable is equal to 1 if there is a measurement error (false negative or false positive) or 0 otherwise. A set of covariates expected to influence measurement methods are used as independent variables. Statistical analysis was performed in R version 3.3.1 (R Development Core Team 2015).

## Results

### Distribution of Measurement Methods

**Fig. 3:**
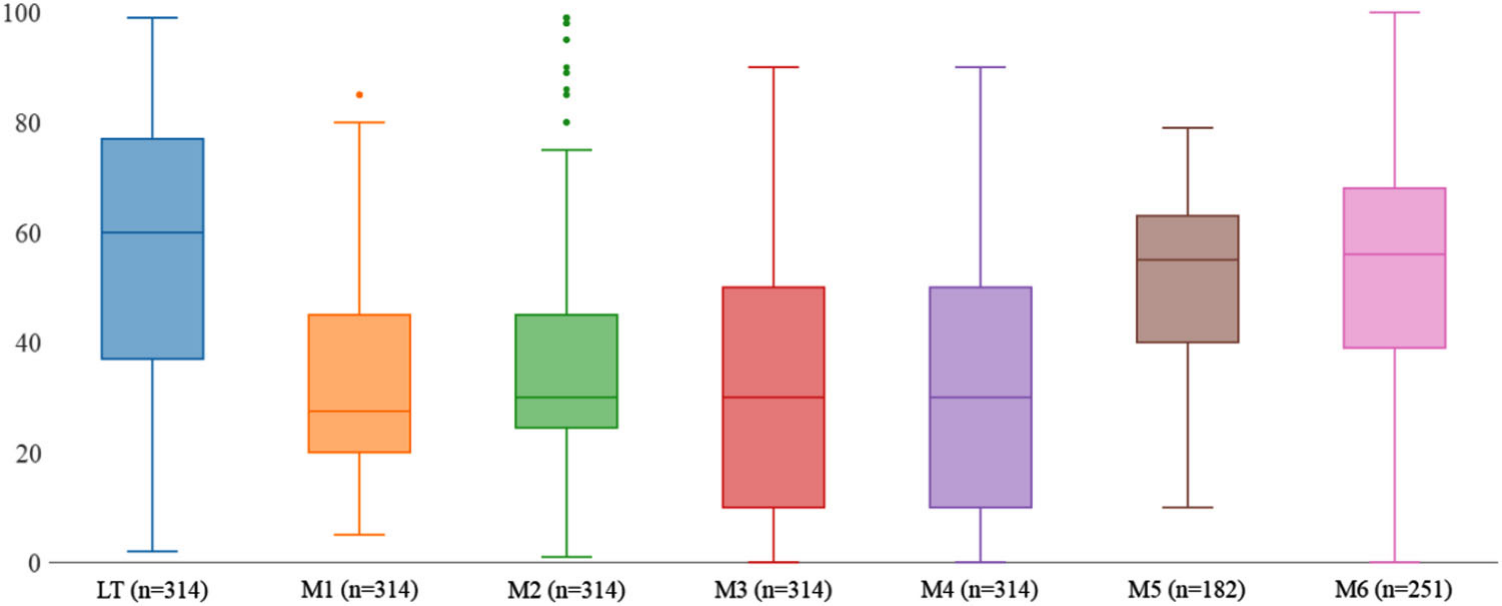
Boxplots of mean crop residue coverage (%) between the benchmark (LT) and the six alternative measurement methods

In Fig. 3, we present box plots for the different measurement methods. We observe that the line-transect reference measure shows a full range of possible values for crop residue coverage, with a distribution ranging from 0 to 100. The median residue cover measured by the LT is 60%.

When considering the median, a major gap exists between the line-transect benchmark and all four survey-based methods (M1–4). The interviewee estimation method (M1) underestimates residue cover by 30 percent points. Having the enumerator visit the plot (M2) only performs slightly better than M1 (+2.5 percent). Surprisingly, collecting data through a visual-aid protocol (M3) does not seem to provide better estimates of the mean distribution than methods based on un-aided responses (M1). The median distribution of the drone method (M5) appears closer from the line-transect method (−5 percent). However, data collected with this method appear relatively concentrated in terms of overall distribution, suggesting a lack of precision at low and high ends of the spectrum of residue cover. The remote sensing method (M6) has a median and overall distribution that appear to best match the LT benchmark.

### How Well Did The Measures Correlate?

Although the overall distribution of each method across the sample provides an interesting first check, we are interested in the correlations between methods at the plot level (Table 3). The interviewee and enumerator visual-aids (M2 and M4) showed the highest coefficients with the line-transect benchmark (0.73 and 0.76). Correlations were lower for interviewee and enumerator estimations (0.60 and 0.57), as well as for the remote sensing method (0.57). Counter-intuitively, the drone image processing method has a negative coefficient of −0.25.

**Table 3:** Spearman’s rho correlations between crop residues coverage measurement methods

	LT	M1	M2	M3	M4	M5	M6
LT	1						
M1	0.60	1					
M2	0.73	0.68	1				
M3	0.59	0.76	0.55	1			
M4	0.76	0.62	0.75	0.6	1		
M5	−0.25	−0.32	−0.16	−0.26	−0.28	1	
M6	0.57	0.42	0.39	0.42	0.47	0.09*	1

All correlations significant at the *p* < 0.001 level at the exception of *, not significant

We also observe correlations between interviewee’s perceptions (M1 and M3) and enumerator’s perceptions (M2 and M4). This demonstrates coherence between respondent’s answers, whether it is the interviewee or the enumerator.

**Fig. 4:**
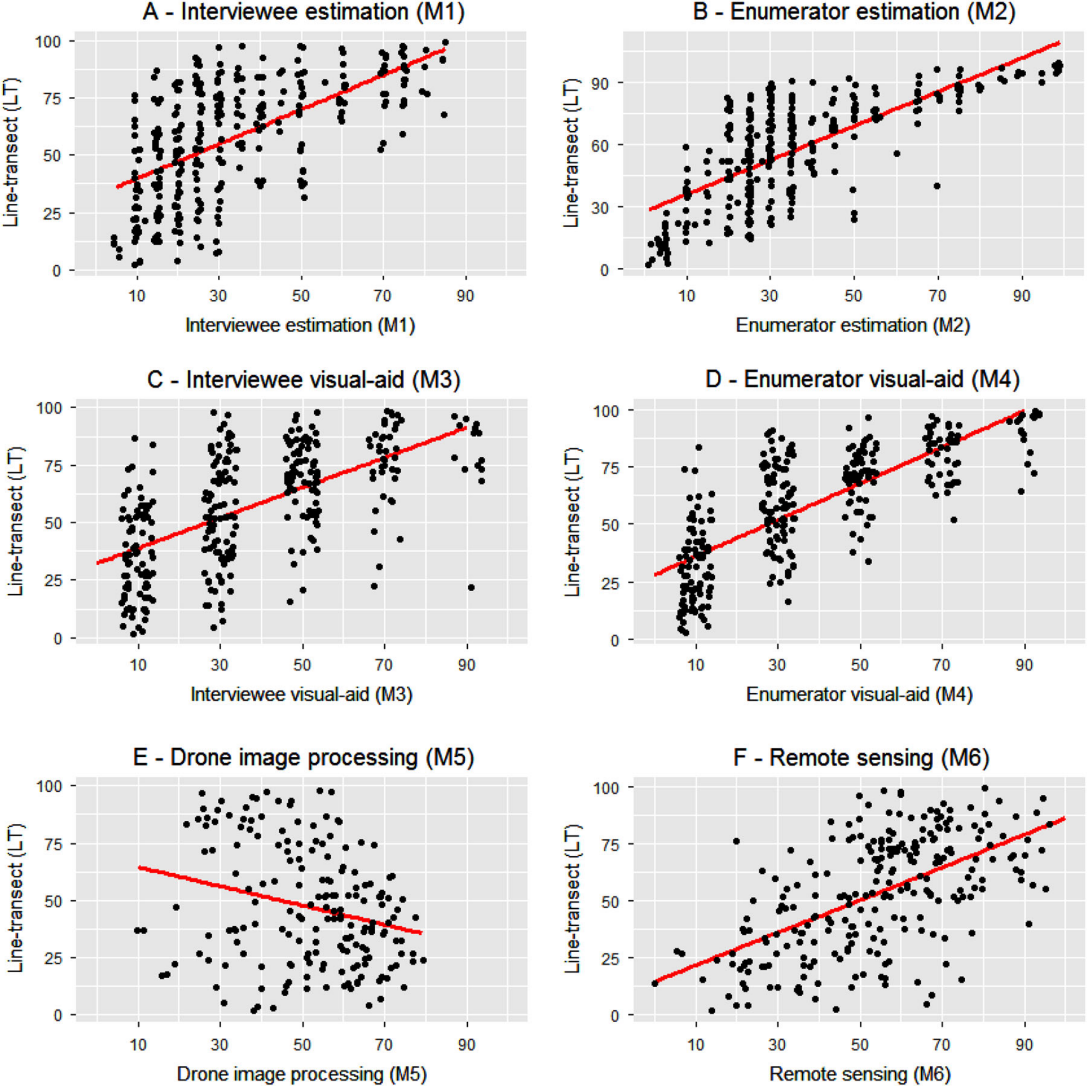
Scatterplots of the six alternative measurement methods against the LT benchmark

Scatter plots of the six alternative measurement methods are plotted against the LT benchmark in Fig. 4. The *red line* indicates the linear fit. The underestimation of residue cover by methods M1 to M4 is confirmed at the plot level. The interviewee estimation shows under-reporting for all levels of coverage. A high level of measurement errors in the 10–30% range appears particularly problematic. This pattern is also observed in the 20–35% range in the case of enumerator estimation. However, M2 appear less likely to under-report plots with a high level of residue. Compared to the respondent visual-aid method (M3), measures obtained by enumerators (M4) have more scattered values at the 30% cover and beyond. The negative correlation of M5 does not seem to follow a consistent pattern. However, we observe that plots that are scattered along the 45° line are more often vertisols and have maize residues. These two field characteristics are likely to facilitate the segmentation process of soil and residues. The remote sensing method (M6) tends to slightly underestimate low residue coverages while slightly overestimate fields with higher amounts of residues.

### How does Measurement Error Affect Adoption Estimates, by Method?

**Fig. 5:**
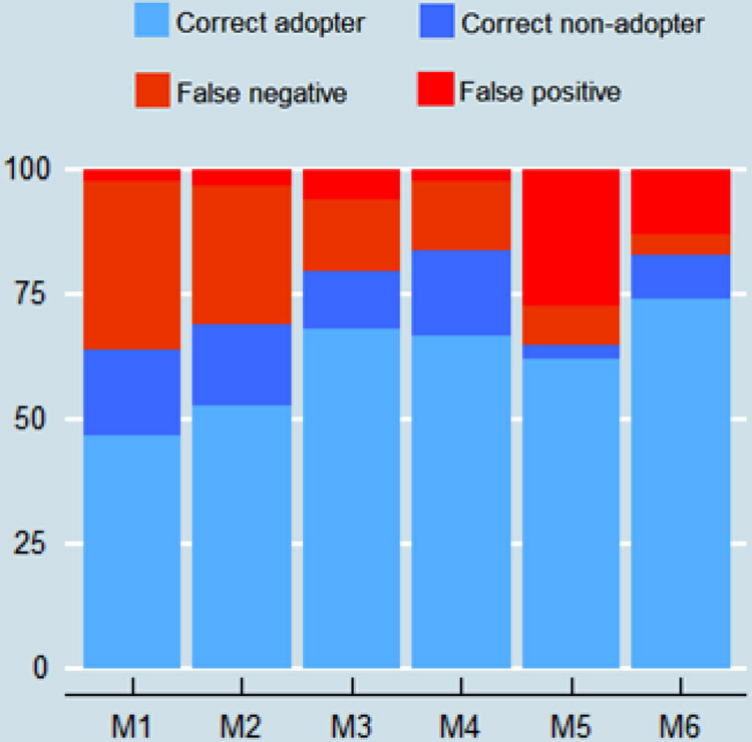
Adoption false reporting of a minimum 30% crop residue cover

In this section, we compare how adoption estimates may vary among crop residue coverage measurement methods. To what extent does the measures classify plots similarly? Figure 5 shows that all methods perform differently when using a categorical threshold. At the 30% threshold, the highest accuracy rates are provided by enumerator with visual-aid (84%), the remote sensing method (83%) and interviewee with visual-aid (80%). Visual-aid methods have a higher level of false negative while remote sensing have a majority of false positive. While the remote sensing method was not as strongly correlated in continuous quantitative analysis as other methods, the categorical comparison delivers a different picture.

**Fig. 6:**
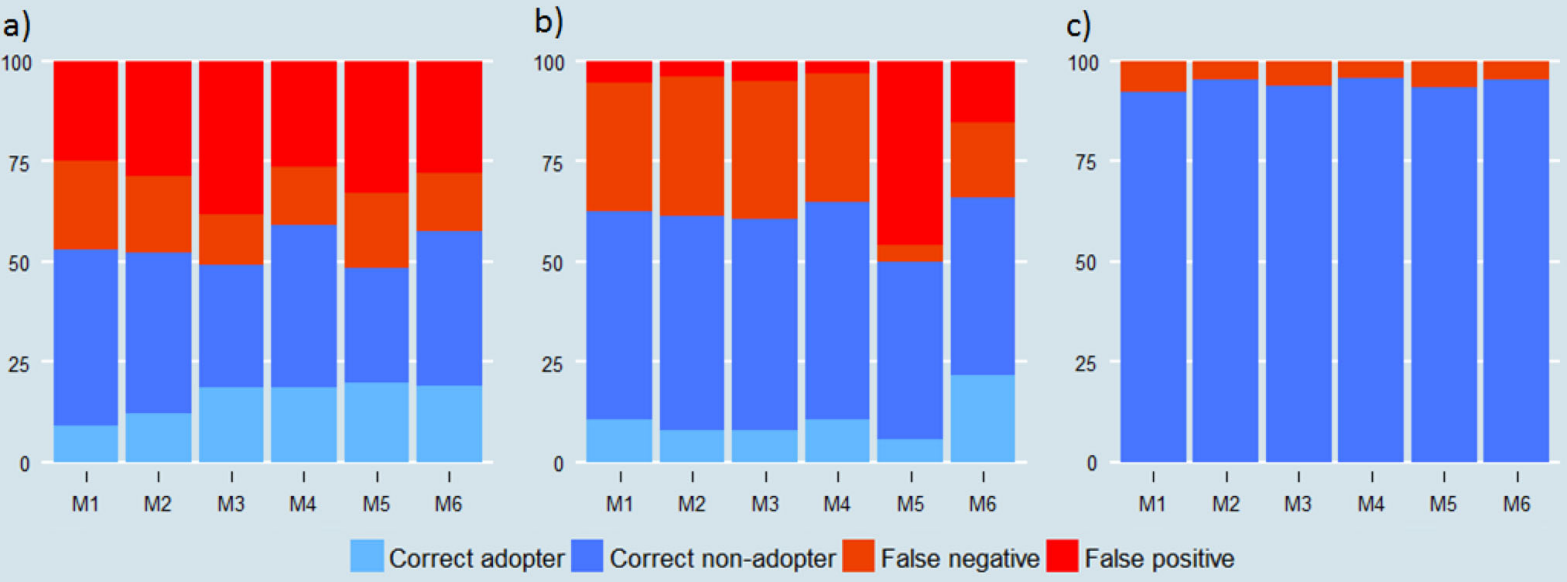
Adoption false reporting of a **a** 30–60% crop residue cover, **b** 60–90% crop residue cover and **c** >90% crop residue cover by method of data collection

Next, we analyzed the extent to which each method over- or under- reports adoption across the FAO AQUASTAT categories of 30–60, 60–90, and >90% ground cover. The most consistent message that comes from Fig. 6 is that none of the measurement methods succeeds in collecting highly accurate data on a categorical scale. Despite the fact that M4 and M6 performed relatively better (Figs. 6a, b), there are still substantial measurement errors. Concerning the identification of a >90% cover, we note that even though 4% of plots were classified as such by the line-transect, none of the alternative measurement methods was actually able to correctly classify these fields.

### Determinants of Measurement Errors

As a final analysis, the determinants of measurement errors for each method are estimated. Since a 30% minimum coverage is regarded as a threshold by conservation agriculture principles, the dependent variable is a binary outcome equal to 1 if there is a measurement error (false negative or false positive) or 0 otherwise (correct adopter or correct non-adopter). Linear probability models are used to estimate the likelihood of measurement error for each method. The models include various potential determinants of measurement errors, related to household and plot characteristics.

Household characteristics are likely to influence survey-based methods relying on the respondent’s self-reporting. It is expected that the ability to estimate crop residue cover may decline with age of the respondent and that education may also improve self-reporting accuracy, especially in the case of percentage estimations. Having participated on a training in crop residue is included as an explanatory variable as trained respondents may already be familiar with crop residue management principles. In other contexts, farm size has been associated with the quality of interviewee’s responses (Kondylis et al. 2015) and this variable was also included. Herd size (in Tropical Livestock Units) and the number of mobile phones in the household are used as proxies of household wealth. Since distance to the plot may decrease the frequency of visits from the interviewee, it is also hypothesized that distance to the plot could be significant in determining measurement errors.

Several plot characteristics were also included as covariates. It is recognized in the remote sensing literature that landscape components greatly impact the ability of microwave signals to detect crop residues (McNairn et al. 2001; Zheng et al. 2014). We therefore control for residue type, soil type and the amount of rocks (subjectively assessed by enumerators)—characteristics that may affect measurement errors of all methods. Field size and slope are included on the grounds that large and flat fields may result in larger measurement errors for survey-based methods and smaller errors for aerial-based methods.

The modeling results are presented in Table 4. Among survey-based methods, the strongest correlate of measurement error is field size (highly significant in 3 out of the 4 methods). This confirms the intuition that human perceptions could limit accurate residue coverage estimation. Field slope significantly decreases the likelihood of measurement errors among the M1 and M4 methods, which is consistent with the idea that more sloped fields provide a more favorable angle for someone to view the entire field and accurately estimate residue cover. The positive effect of distance on measurement error is also intuitive; compared with closer plots, more distant plots may not be visited as often or receive the same intensity of management attention. However, contrary to our expectations, aerial methods do not perform better on larger fields.

**Table 4:** Linear probability models of the factors affecting the probability of false reporting adoption (minimum 30% coverage)

	M1	M2	M3	M4	M5	M6
*Household characteristics*						
Sex of the head (Ref = Male)	−0.07		0.00			
Age of the head	0.00		0.00			
Years of education	0.01		0.01			
Training on crop residue management	−0.06		0.05			
Total farm size	0.03**		0.00			
Herd size (in TLU)	0.00		−0.01			
Number of mobile phones	0.01		0.03			
Distance from the field	0.00*		0.00**			
*Field characteristics*						
Field size	0.00***	0.00	0.00***	0.00**	0.00	0.00*
Barley residues	−0.07	−0.03	0.03	−0.05	0.10	−0.07
Maize residues	0.02	0.04	0.3****	0.15**	0.00	0.3***
Wheat residues	0.11	0.10	0.13*	0.11*	−0.26***	−0.04
Cambisol soil type	−0.21**	−0.12	−0.03	−0.08	0.28	0.1
Luvisol soil type	−0.36***	−0.11	−0.12	−0.14**	0.25*	0.08
Vertisol soil type	−0.08	−0.05	−0.07	−0.16***	−0.10	0.24***
>20% rocks	−0.04	0.04	0.02	0.03	0.02	−0.05
Slight slope	−0.14*	−0.11	−0.07	−0.14***	0.14	−0.04
Steep slope	−0.08	−0.03	−0.17	−0.23**		−0.05
Intercept	0.22	0.33***	0.07	0.19**	0.46***	0.00
*N*	314	314	314	314	182	251
Adjusted *R* ^2^	0.17	0.01	0.17	0.15	0.07	0.25

*, **, *** Statistically significant at the 0.1, 0.05, and 0.01 level respectively

We found almost no impact of household characteristics on interviewee’s answers accuracy. Consistent with Kondylis et al. (2015), farm size is the only parameter to be significantly associated with measurement errors. Surprisingly, years of education does not affect the accuracy of answers. We were unable to control for differences in enumerator’s abilities in M2 and M4 owing to very small number of enumerators used so several unobserved factors may thus bias the results.

Crop residue and soil type also affect measurement errors of survey-based and aerial-based methods. The accuracy of enumerators’ answers was lower, with more errors reported, in the case of maize and wheat residue (M3 and M4). However, wheat residues were better captured with the drone image processing method, while maize residues are associated with greater measurement errors using the remote-sensing method (M6). Luvisol (*red*) and vertisol (*black*) soil types allow respondents and enumerators to better distinguish residue cover (M1 and M4). The opposite is true for aerial-based methods where luvisol soil types increase the likelihood of measurement errors with the drone image processing method; and vertisols soils results in larger errors with the remote sensing method. This result is likely due to the higher moisture content of clay, dark soils, which may have affected spectral reflectance.

## Discussion and Conclusion

Conservation agriculture has received considerable attention among scholars and policy makers in recent years. However, empirical evidence of large scale adoption and impact has remained scarce and considerable variation exists in adoption estimates (Andersson and D’Souza 2014; Stevenson et al. 2014). Despite the important implications for policy and resource allocation, very few studies have examined the accuracy of crop residue coverage—a key element of conservation agriculture.

In this article, the primary goal is to advance the discussion by presenting results of a methodological validation exercise in which six alternative methods of crop residue coverage measurement were tested among the same sample of rural households in Ethiopia, and compared against a LT benchmark. This article attempts to fill an academic and policy demand through an examination of low-cost methods for capturing field crop residue coverage information in a continuous and categorical form.

What stands out from the results is that survey-based methods tend to underestimate crop residue coverage across fields and this pattern was prevalent among interviewee’s responses as well as enumerators’ observations. This finding could be explained by a context of communal grazing where respondents tend to think that field residue cover is lower than it actually is. However, this explanation is not convincing for enumerators, where this result suggests inherent limits to human perceptions. Thus the methods that are often employed by National Statistical Offices would be misleading in measuring soil conservation practices. The measurement error is more serious when estimating percentages.

Despite the presence of measurement errors in all alternative methods, this research has delivered a clear ranking of measurement options. The visual-aid method yields the most accurate estimates of the true distribution as well as adoption of a minimum 30% cover. Thus, from low-cost alternative methods of data collection, results support a wider use of visual-aid protocols as an alternative to self-reported percentage cover estimations. The visual-aid protocol employed in this experiment is presented in Appendix A. It is also noteworthy that none of the alternative measurement methods were able to estimate FAO’s AQUASTAT categories of 30–60, 60–90, and >90% ground cover. Thus, the reporting of these more detailed statistics would call for more exploration.

Concerning aerial-based methods, our attempts were constrained by the necessity to identify low-cost and easy to implement solutions. This should be kept in mind, and certainly weighs on the accuracy of the results that were obtained. In contrast with results from Woebbeck et al. (1995) and Asadi and Jafari (2011) where field images were obtained at a distance of about 2.4 m height from the ground, under controlled conditions, results from the drone image processing method in this experiment were not satisfactory. In comparison with ground field images, the use of aerial images taken by drones certainly introduces additional sources of errors. An examination of aerial images taken at different altitudes has revealed modifications in the color of soil components. This suggests that the sensitivity of camera sensors to the prevailing lighting conditions may explain the inability of the segmentation algorithm to discriminate the crop residues from the soil. Given their potential to monitor adoption of agricultural technologies, research on the use of drones should be pursued; more sophisticated technologies may allow for higher accuracy of image-processing methods.^6^

In binary outcome estimates of whether a plot meets a minimum 30% residue cover threshold, the remote sensing method performed well, with an 83% accuracy rate. Given the fact that Landsat 8 images have a fairly coarse resolution sensor (30 m/pixel) and that the NDTI index was not calibrated, this result is very encouraging. Indeed, the relatively large pixel size of Landsat 8 and the relatively small field sizes utilized in the study may generate “mixed-pixel” situations where only portions of a 30 m pixel actually fall over a given field. Therefore, some of the NDTI calculations could be reporting measures of residue that may be outside the field boundaries. Using a higher resolution sensor for this analysis would help alleviate this issue, though some “mixed-pixels” are almost always inevitable with remotely sensed imagery from a space platform. In addition, results suggest that the NDTI could gain in accuracy by research focusing on the effect of field variations (crop residue type, moisture, soil type/color) on spectral reflectance. The recent availability of high quality, freely available satellite data (Landsat 8, Sentinel-1), as well as the prospect of multi-sensors combining multispectral and hyperspectral data gives plenty of room for optimism.

Some limitations of this study should be acknowledged. First, the sample is representative of a few enumeration areas only. Since measurement errors are likely to be different in different socio-economic and agro-ecological contexts, the replication of this methodological experiment in different setting should be encouraged. Second, some aspects of crop residue cover that are important for research could not be explored in this paper. These include the depth of the residue cover as well as the timing of data collection. While farmers in the area are using minimum tillage, uncontrolled grazing limit their ability to implement conservation agriculture. Future studies should take into consideration residue cover measurement over a wider period of time, possibly by looking at several agricultural seasons. In addition, soil moisture and residue moisture content—particularly important for remote sensing estimation—could not be explored in this paper.

However, results reported here can potentially serve as guidance for survey practitioners and have implications for future household surveys. Survey-based analyses of adoption relying on self-reported estimation should be taken with caution and we advocate a wider use of visual-aid protocols (see Fig. A.1) for collecting survey-based data on natural resource management practices. Although aerial-based methods provide several promising research paths for the improvement of agricultural data, this experiment suggests that crop residue cover can be measured on the ground. We hope that these results will be taken up in future questionnaire design by National Statistical Institutes and researchers working on adoption and measurement of impact of conservation agriculture.

## Electronic supplementary material

Supplementary Material

## Footnotes

1 For statistical institutes, a relevant measure of adoption would be based on the criterion that the method used is at least reliable to determine the presence of a minimum 30% field coverage.

2 ESS is the Living Standards Measurement Study—Integrated Surveys on Agriculture (LSMS-ISA) program in Ethiopia.

3 Farmers generally have multiple uses for crop residues.

4 In Afaan Oromo language, maize residue are named “Agada”, wheat and barley residues “Galabaa” and teff residues “Qaranii”.

5 GPS coordinates for each corner of the plot was taken with a <5 m accuracy.

6 Piloting and picture-taking are fairly easy procedures that can be performed by enumerators with few hours of training. During the survey, the camera apparels ceased to function, possibly due to the effect of dust on electronic components. It should also be noted that given the recent introduction of these apparels, obtaining legal authorizations in Ethiopia, in the absence of a legal framework, was a tedious process.
