# Supplementary figures and images for: On the Ground or in the Air? A Methodological Experiment on Crop Residue Cover Measurement in Ethiopia
Source: Environ Manage. 2017 Jun 8;60(4):705–16. doi: 10.1007/s00267-017-0898-0 (PMC5602098; doi:10.1007/s00267-017-0898-0)

Figure A.1 Visual-based protocol showing crop residue coverages at the 0, 10, 30, 50, 70 and 90 % levels


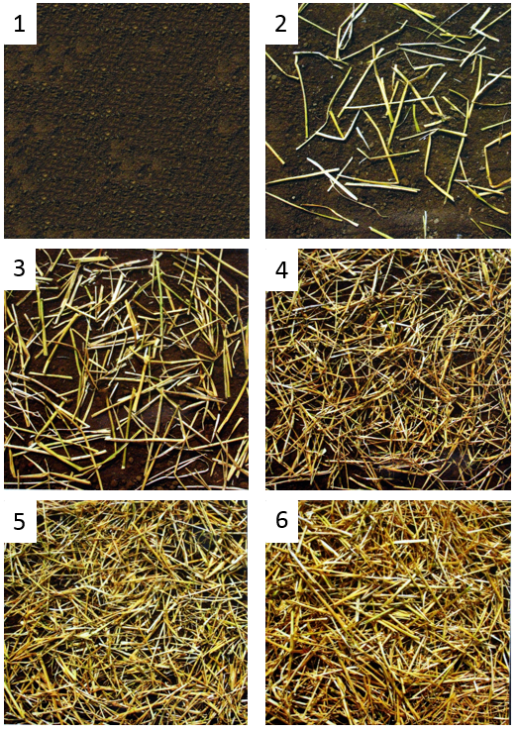

Supplement: Supplementary file 1 — Supplementary Material [file 267_2017_898_MOESM1_ESM.docx]
